# An Atypical Presentation of Giant Cell Arteritis in an Elderly Patient: Diplopia and Fever Masked by Pneumonia

**DOI:** 10.7759/cureus.95078

**Published:** 2025-10-21

**Authors:** Aleena Anna George, Solomon Yimer

**Affiliations:** 1 General Internal Medicine, Macclesfield District General Hospital, Macclesfield, GBR

**Keywords:** atypical pneumonia, binocular diplopia, fever, giant cell arteritis (gca), large vessel vasculitis

## Abstract

Giant cell arteritis (GCA) is a chronic vasculitis typically affecting individuals over 50 years of age, often presenting with headache, jaw claudication, scalp tenderness, or visual symptoms. We report an 86-year-old male with psoriasis and hypertension who presented with cough, fever, night sweats, headache, and binocular diplopia, initially managed as pneumonia and possible stroke. Despite antibiotics, his fever and ocular symptoms persisted. Elevated inflammatory markers and temporal artery ultrasound confirmed GCA. Prompt corticosteroid therapy led to clinical improvement. This case highlights an atypical presentation of GCA masked by respiratory infection, emphasizing the importance of early suspicion to prevent irreversible vision loss.

## Introduction

Giant cell arteritis (GCA) is a granulomatous large-vessel vasculitis primarily affecting individuals over 50, classically presenting with temporal headache, jaw claudication, scalp tenderness, and visual disturbances, often accompanied with elevated inflammatory markers and sometimes polymyalgia rheumatica [1]. However, there is growing recognition that GCA may manifest atypically, with non-specific or constitutional symptoms such as fever of unknown origin (FUO), malaise, weight loss, or even stroke, complicating and delaying diagnosis [2,3]. Such presentations may mask underlying vasculitis especially in elderly patients, where overlapping infections or comorbid conditions blur the clinical picture.

Diagnostic sensitivity of standard investigations (temporal artery biopsy, ultrasound) can be lower in these atypical cases, and negative imaging or biopsy does not always exclude the disease [3,4]. Early suspicion and prompt empiric treatment are critical, since delay in diagnosis may lead to irreversible complications, particularly vision loss. This report describes the case of an elderly patient whose presentation was initially thought to be that of a chest infection; however, persistent systemic and neurological findings ultimately led to the diagnosis of GCA, underscoring the importance of considering GCA in atypical settings.

## Case presentation

An 86-year-old male presented to the emergency department with a three-week history of persistent non-productive cough and a three-day history of night sweats, fever, and chills. Around the same time, he developed a frontal headache, described as moderate in intensity, scoring 6 out of 10 on the pain scale, non-radiating, and without any particular exacerbating or relieving factors. There was no associated photophobia, phonophobia, nausea, or vomiting. He also reported the sudden onset of horizontal binocular diplopia when looking at distant objects. His past medical history included hypertension, psoriasis, and an inguinal hernia repair. He was not on immunosuppressive therapy. He had smoked in the past with a cumulative history of 0.625 pack-years but had quit more than 50 years earlier. He consumed one glass of wine daily. He denied recent weight loss, rashes, diarrhea, dysuria, palpitations, or chest pain. There was no significant family history of autoimmune or rheumatological conditions.

On examination, he was alert and oriented with a temperature of 38 °C, blood pressure of 138/76 mmHg, heart rate of 88 beats per minute, respiratory rate of 20 per minute, and oxygen saturation of 97% on room air. He appeared frail but was not in acute distress. General examination showed warm peripheries, no peripheral edema, and no rashes. His temporal arteries were palpable bilaterally and not tender or thickened. Respiratory examination revealed coarse crepitations over the right lung base. Cardiovascular examination showed an ejection systolic murmur, grade II/VI, without radiation. Peripheral pulses were intact and symmetrical. The abdominal examination was unremarkable, with a soft and non-tender abdomen and no organomegaly. Neurological examination demonstrated horizontal binocular diplopia on lateral gaze, consistent with abducens nerve involvement. There were no other cranial nerve deficits, no motor or sensory deficits, and no meningeal signs.

**Figure 1:**
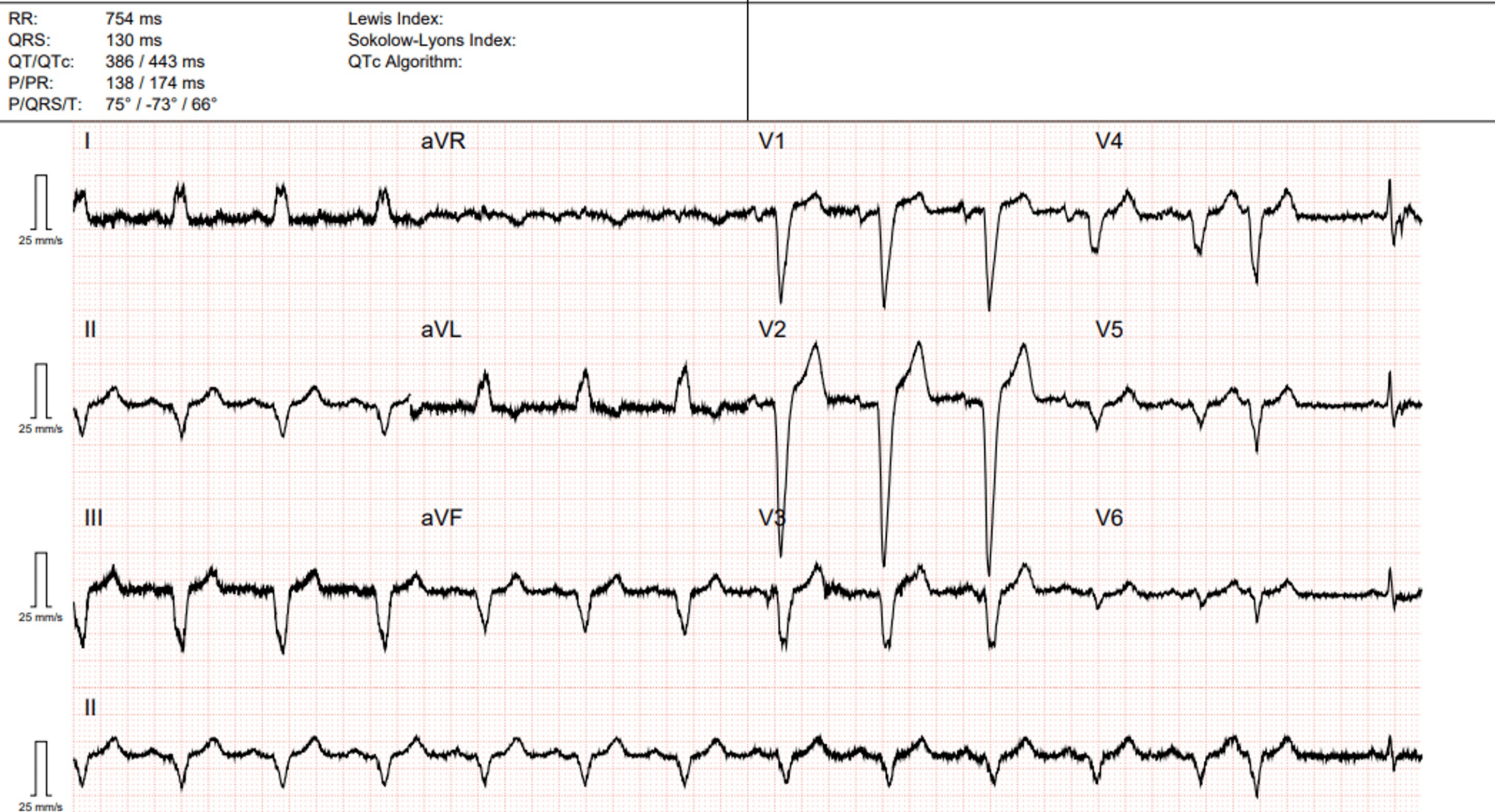
ECG of the patient showing left bundle branch block.

**Figure 2:**
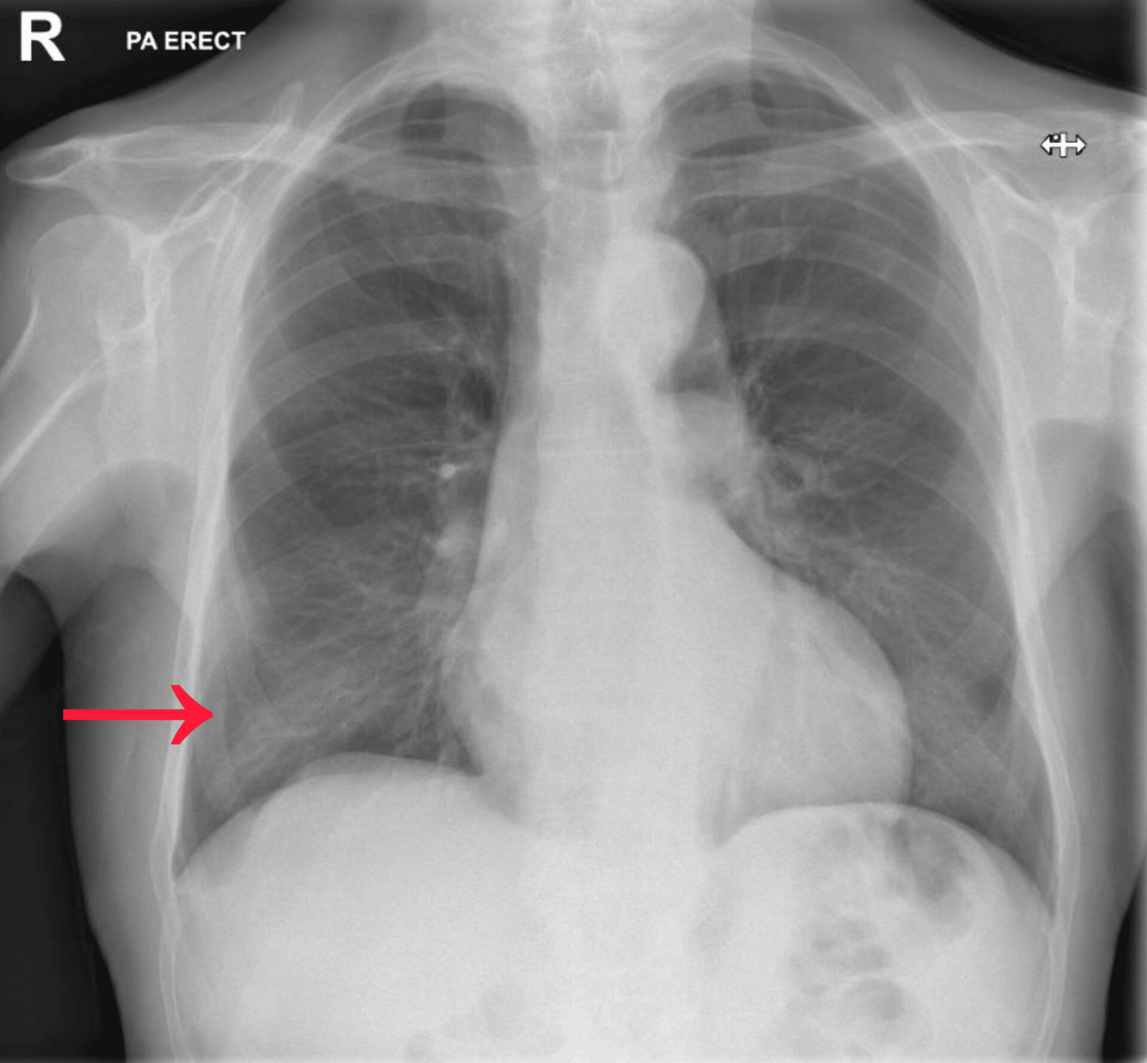
Chest X ray of the patient showing right lower lobe consolidation.

**Figure 3:**
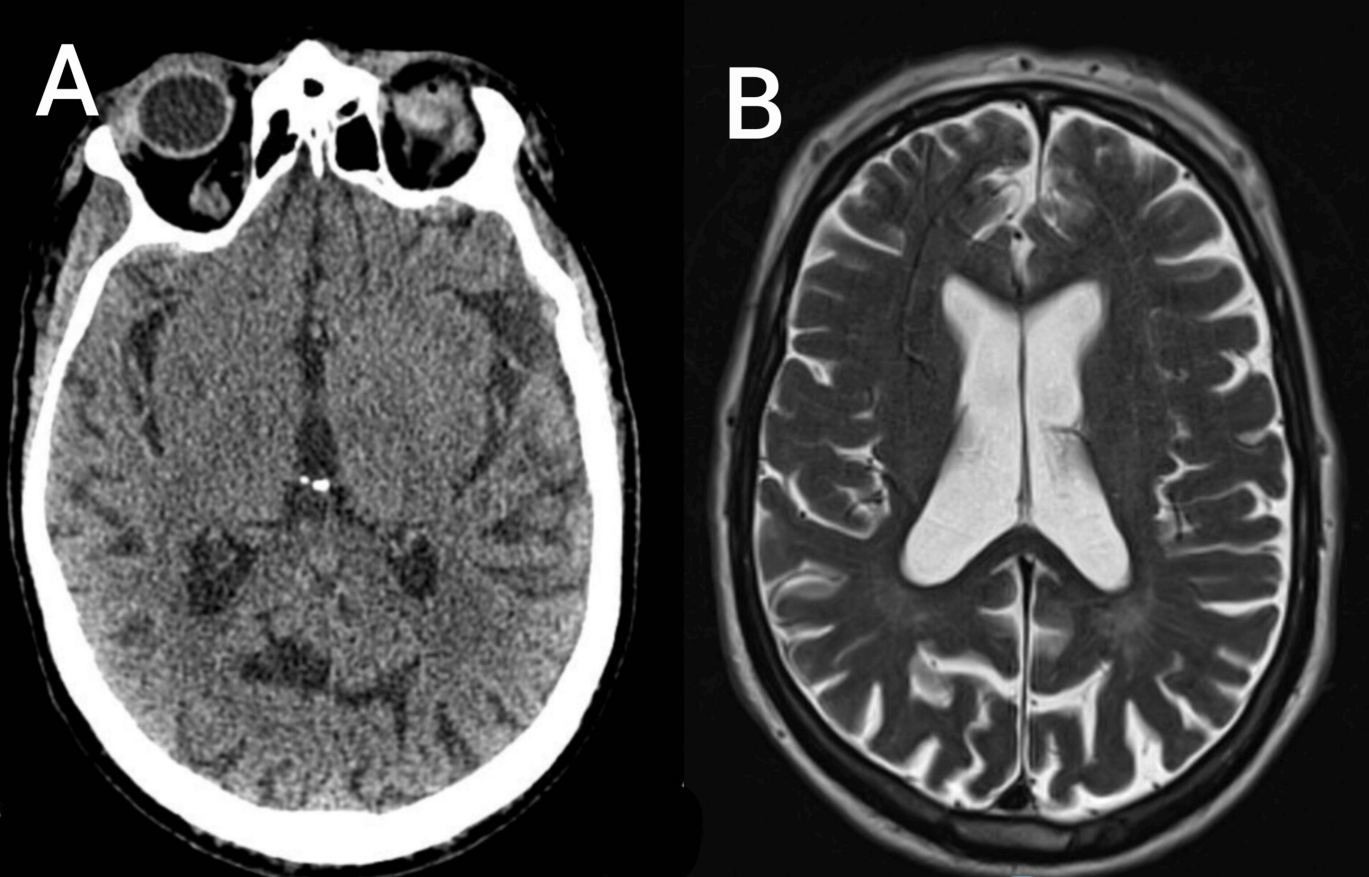
CT and MRI brain of the patient showing no significant abnormality. (A) A section of CT brain of the patient; (B) A section of MRI brain of the patient.

Initial laboratory investigations revealed white cell count of 13.5 × 10⁹/L with neutrophils 10.53 × 10⁹/L and lymphocytes 1.08 × 10⁹/L. The C-reactive protein was markedly elevated. Renal and liver function test were normal (Table 1). Electrocardiogram showed left bundle branch block (Figure 1). Chest X-ray demonstrated right lower lobe consolidation (Figure 2). A non-contrast CT scan of the head and MRI brain did not reveal any acute intracranial abnormality (Figure 3).

**Table 1:** Laboratory values of the patient. ALT: alanine amino transferase; AST: aspartate amino transferase; SGPT: serum glutamate-pyruvate transaminase; SGOT: serum glutamic-oxaloacetic transaminase

Test Name	Patient Value	Reference Range
Hemoglobin	122 g/L	Males (130–170 g/L)
Total Leukocyte Count	13,500 /µL	4000-11000 /µL
Platelet Count	17,9000 /µL	150,000–450,000 /µL
C-Reactive Protein (CRP)	179 mg/L	<5 mg/L
Sodium	137 mmol/L	135-145 mmol/L
Potassium	4.3 mmol/L	3.5–5.0 mmol/L
Chloride	101 mmol/L	98–106 mmol/L
Urea	5.6 mmol/L	2.5 to 7.1 mmol/L
Creatinine	89 µmol/L	53-97 µmol/L
Total Bilirubin	13 µmol/L	5.1 and 17.0 µmol/L
ALT (SGPT)	27 U/L	10–40 U/L
AST (SGOT)	24 U/L	10–40 U/L
Alkaline Phosphatase	94 U/L	40–129 U/L

The working diagnosis at admission was community-acquired pneumonia with a possible posterior circulation stroke given his acute diplopia and vascular risk profile. He was started empirically on intravenous piperacillin-tazobactam for pneumonia and was loaded with aspirin 300 mg daily after hemorrhage was excluded. By the second day, given the ongoing fever, alternative diagnoses such as meningitis were considered. Antibiotics were switched to intravenous ceftriaxone. A lumbar puncture was performed, which showed cerebrospinal fluid glucose of 3.6 mmol/L (2.5-4.5 mmol/L), protein of 0.4 g/L (0.15-0.45 g/dL), white cells of 4/mm³ (0-5 /mm³), and no red cells (Table 2). No organisms were detected. Blood cultures remained negative.

**Table 2:** CSF values of the patient. CSF: cerebrospinal fluid

CSF Test Name	Patient Value	Reference Range
Glucose	3.6 mmol/L	2.5–4.5 mmol/L
Protein	0.4 g/dL	0.15–0.45 g/dL
White Cell Count	4 /mm³	0–5 /mm³
Red Cell Count	0 /mm³	0 /mm³

On the third day, an MRI of the brain was carried out to exclude small posterior circulation infarcts or meningeal disease, but the scan was normal. With no clear evidence of central nervous system infection, antibiotics were de-escalated to oral doxycycline. On the fifth day, his cough had improved and chest was clear on auscultation, with no oxygen requirement. Despite this, he continued to have fever spikes and his headache persisted, along with binocular diplopia. His inflammatory markers continued to rise, with C-reactive protein (CRP) now at 309 mg/L, while his white cell count was 11.9 × 10⁹/L. Given the absence of ongoing infective source and persistent systemic inflammation, the differential diagnosis was broadened. At this point, systemic vasculitis was considered. Erythrocyte sedimentation rate was 44 mm/hour. A vasculitis screen including antinuclear antibody (ANA), antineutrophil cytoplasmic antibodies (ANCA), and complement levels was negative. In view of the continuing fever, headache, and diplopia, he was started on oral prednisolone 40 mg daily with gastric protection.

**Figure 4:**
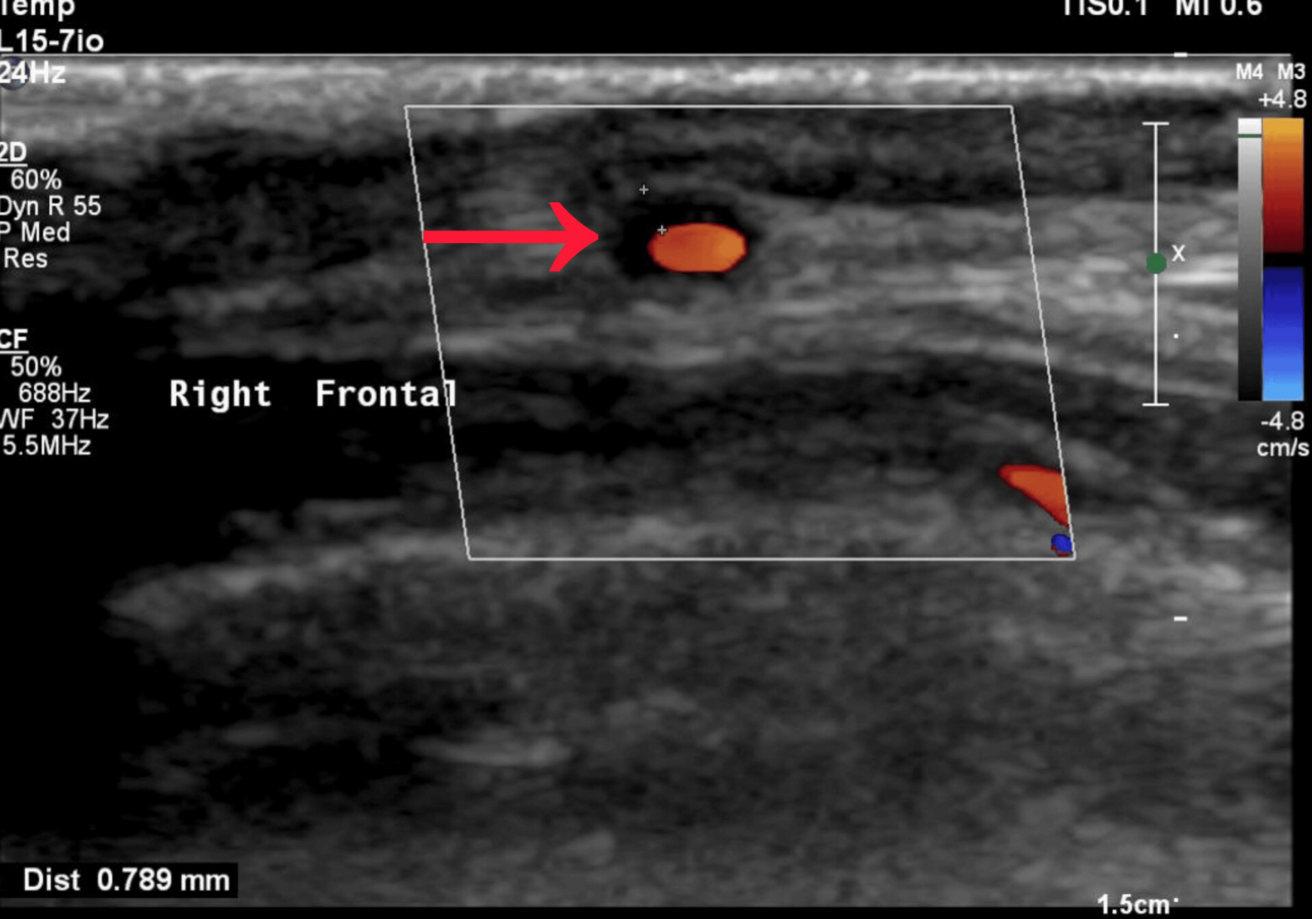
Ultrasound Doppler of temporal artery showing halo sign (dense vessel wall thickness with a narrow lumen).

By the seventh day, he was reviewed by rheumatology. The Giant Cell Arteritis Probability Score (GCAPS) [5] was calculated as 20, which fell into the category of high likelihood of disease. Based on his presentation and high probability score, along with persistently elevated inflammatory markers, infection was deemed unlikely, and corticosteroid therapy was escalated to prednisolone 60 mg daily. On the eighth day of admission, inflammatory markers showed improvement, with CRP falling to 251 mg/L. The patient’s headache subsided, fever settled, and diplopia improved gradually. Antibiotics were discontinued at this stage. A bilateral temporal artery ultrasound was performed, which demonstrated a hypoechoic halo sign localized to the right frontal branch, consistent with temporal arteritis (Figure 4).

A biopsy was not done from the temporal artery as the diagnosis was obvious from clinical and radiological findings. The patient was discharged on a tapering regimen of oral prednisolone, starting at 60 mg daily for two weeks, reducing by 10 mg every two weeks until reaching 30 mg daily, with a slower taper thereafter as per rheumatology guidance. He was also prescribed bone protection with calcium, vitamin D, and bisphosphonate prophylaxis. On follow-up at four weeks, he reported complete resolution of fever and significant improvement of headache, with near resolution of diplopia. His CRP had normalized to below 10 mg/L. At three months, he remained stable without recurrence of systemic or neurological symptoms. The steroid taper was ongoing under specialist supervision, and he had no evidence of steroid-induced complications.

## Discussion

GCA is the most common form of primary systemic vasculitis in adults, typically affecting individuals over the age of 50 years. It characteristically involves large- and medium-sized arteries, with the temporal arteries being the most frequent site. Classical manifestations include headache, jaw claudication, scalp tenderness, visual disturbances, and constitutional symptoms such as fever, malaise, or weight loss [1,2]. However, the clinical spectrum is wide, and many patients present with atypical features that can obscure the diagnosis. In such scenarios, early recognition and prompt initiation of corticosteroid therapy remain essential to prevent irreversible complications, particularly permanent visual loss [3,4]. The patient described in this report presented with cough, fever, and night sweats, initially suggesting an infective etiology. Chest radiography demonstrated right lower lobe consolidation, and a working diagnosis of community-acquired pneumonia was made. Although his chest symptoms improved with antimicrobial therapy, fever persisted and inflammatory markers continued to rise. This incongruity between clinical response and laboratory trends was a significant clue that infection alone was not the underlying cause. Such systemic inflammatory responses are frequently observed in GCA and can often be misinterpreted as infection, particularly in elderly individuals who are prone to both conditions [6].

The presence of binocular diplopia was another striking feature. Ocular involvement in GCA is well documented, yet diplopia is relatively uncommon, occurring in approximately 5% of cases [7]. It usually results from ischemia of the extraocular muscle nerves, most commonly the abducens nerve, though third and multiple cranial nerve palsies have also been described [8]. In this patient, diplopia persisted despite resolution of chest infection, thereby further supporting the presence of an underlying vasculitic process rather than an isolated respiratory illness. Importantly, visual acuity was preserved, which may be attributed to the timely initiation of corticosteroid therapy. Several case reports have described diplopia as an early or predominant manifestation of GCA. A study reported an 85-year-old man presenting with dizziness and diplopia as the leading symptoms, later diagnosed as GCA [8]. Another study documented an 77-year-old patient with fatigable ptosis and diplopia, again preceding the development of classical cranial symptoms [9]. Another report highlighted a case with bilateral internuclear ophthalmoplegia (INO), further emphasizing the diverse ocular manifestations [10]. A similar report, described GCA presenting as bilateral INO reinforcing that isolated ocular motor symptoms can be a harbinger of the disease [11]. Our case shares similarity with these reports in that diplopia was a key presenting symptom, but it differs in being initially masked by coexistent pneumonia, which delayed the consideration of vasculitis. This interplay of infection and systemic inflammation makes the case unique and educational for clinicians.

The diagnostic workup in this case followed a systematic approach. Stroke and central nervous system infection were ruled out with brain imaging and lumbar puncture, respectively. Persistently elevated CRP despite antibiotic therapy guided clinicians toward a non-infective inflammatory process. The diagnosis of GCA was subsequently supported by elevated erythrocyte sedimentation rate (ESR), rapid symptomatic improvement with corticosteroids, and the presence of the “halo sign” on temporal artery ultrasound. Non-invasive vascular imaging, particularly duplex ultrasonography, is increasingly recognized as a first-line tool in suspected GCA, with high sensitivity and specificity in experienced hands [12]. Although temporal artery biopsy has traditionally been considered the gold standard, it is limited by sampling error and procedural delay. The rapid accessibility of ultrasound in our case facilitated timely confirmation. In clinical practice, scoring systems such as the Southend GCA Probability Score (GCAPS) can also assist clinicians in stratifying risk. GCAPS incorporates demographic, clinical, and laboratory parameters and categorizes patients into low-, intermediate-, and high-probability groups. In validation studies, a high GCAPS score has been strongly associated with confirmed GCA [5-7]. In our patient, a score of 20 placed him in the high-probability group, consistent with the final diagnosis. The novelty of this case lies in its presentation. While atypical features of GCA have been reported, the coexistence of presumed pneumonia with persistent systemic inflammation and isolated diplopia as the key neurological manifestation is uncommon. Most previous reports of diplopia in GCA have occurred in the absence of concurrent infection, making this case particularly distinctive. Furthermore, the patient demonstrated marked systemic inflammation, with CRP levels exceeding 300 mg/L, a finding more often seen in severe infections rather than vasculitis. The recognition that such profound elevations can occur in GCA is an important reminder for clinicians to maintain diagnostic breadth.

Therapeutically, the patient responded promptly to corticosteroid therapy, with resolution of fever, headache, and gradual improvement in diplopia. No irreversible visual loss occurred, underscoring the critical role of early treatment. The course also highlights the importance of a careful tapering regimen and the necessity of bone protection in elderly patients receiving long-term corticosteroids.

## Conclusions

This case illustrates an atypical presentation of GCA in an elderly patient, initially masked by pneumonia. Persistent fever, headache, and diplopia despite antibiotic therapy prompted consideration of vasculitis. Timely recognition, supported by elevated inflammatory markers and temporal artery ultrasound, allowed prompt corticosteroid initiation, leading to rapid clinical improvement and prevention of visual loss. Clinicians should maintain high suspicion for GCA in elderly patients with unexplained systemic or ocular symptoms to avoid delayed diagnosis and complications.
